# Transgenic Rabbit Models in Proarrhythmia Research

**DOI:** 10.3389/fphar.2020.00853

**Published:** 2020-06-05

**Authors:** István Baczkó, Tibor Hornyik, Michael Brunner, Gideon Koren, Katja E. Odening

**Affiliations:** ^1^Department of Pharmacology and Pharmacotherapy, University of Szeged, Szeged, Hungary; ^2^Department of Cardiology and Angiology I, Heart Center, University of Freiburg, Freiburg, Germany; ^3^Faculty of Medicine, University of Freiburg, Freiburg, Germany; ^4^Department of Cardiology and Medical Intensive Care, St. Josefskrankenhaus, Freiburg, Germany; ^5^Cardiovascular Research Center, Division of Cardiology, Rhode Island Hospital, Alpert Medical School of Brown University, Providence, RI, United States; ^6^Translational Cardiology, Department of Cardiology, Inselspital, Bern University Hospital, Bern, Switzerland; ^7^Institute of Physiology, University of Bern, Bern, Switzerland

**Keywords:** transgenic LQTS rabbit, drug-induced proarrhythmia, proarrhythmia safety screening, K^+^-channel blocker, long QT syndrome, cardiac repolarization reserve

## Abstract

Drug-induced proarrhythmia constitutes a potentially lethal side effect of various drugs. Most often, this proarrhythmia is mechanistically linked to the drug's potential to interact with repolarizing cardiac ion channels causing a prolongation of the QT interval in the ECG. Despite sophisticated screening approaches during drug development, reliable prediction of proarrhythmia remains very challenging. Although drug-induced long-QT-related proarrhythmia is often favored by conditions or diseases that impair the individual's repolarization reserve, most cellular, tissue, and whole animal model systems used for drug safety screening are based on normal, healthy models. In recent years, several transgenic rabbit models for different types of long QT syndromes (LQTS) with differences in the extent of impairment in repolarization reserve have been generated. These might be useful for screening/prediction of a drug's potential for long-QT-related proarrhythmia, particularly as different repolarizing cardiac ion channels are impaired in the different models. In this review, we summarize the electrophysiological characteristics of the available transgenic LQTS rabbit models, and the pharmacological proof-of-principle studies that have been performed with these models—highlighting the advantages and disadvantages of LQTS models for proarrhythmia research. In the end, we give an outlook on potential future directions and novel models.

## Introduction: Proarrhythmia and Drug Development

Proarrhythmia—the triggering of arrhythmias following drug therapy—has been known for many decades as being caused by “anti”-arrhythmic cardiac drugs (Selzer and Wray, 1964; Echt et al., 1991; Waldo et al., 1996). This rare but lethal side-effect of drug therapy, however, is not restricted to anti-arrhythmic drugs, but occurs with a variety of other, non-cardiac drugs (Woosley et al., 1993; Wysowski and Bacsanyi, 1996; Darpo, 2001), and therefore is a major concern for patients, physicians, and the pharmaceutical industry. It has been estimated that around 20–60% of novel chemical entities have the potential to modulate the function of cardiac ion channels and therefore, to disturb normal cardiac electrical function (Danker and Moller, 2014). Depending on the nature of the drug-induced effects on ion channels function, proarrhythmia can be associated with prolongation or shortening of the QT interval (long QT syndrome, LQTS or short QT syndrome, SQTS, respectively) and/or with conduction disturbances.

For most drugs, proarrhythmia is assumed to be based on prolongation of cardiac repolarization as a result of drug-induced inhibition of cardiac potassium currents (mostly *I*_Kr_ current(s)/HERG-channels) (Redfern et al., 2003). This acquired LQTS predisposes to Torsades-de-Pointes (TdP) polymorphic ventricular tachycardia that could lead to ventricular fibrillation and sudden cardiac death (SCD) (Haverkamp et al., 2000; Redfern et al., 2003; Fenichel et al., 2004). Although no less than 2–3% of all marketed drugs have the potential to induce LQTS (Stansfeld et al., 2006), the (documented) incidence of potentially lethal drug-induced TdP is typically very low (1:10,000 for non-cardiovascular drugs) (Yap and Camm, 2003), and therefore very hard to predict reliably (Fenichel et al., 2004). In recent decades, TdP-induced SCD cases were associated with a wide range of commonly used drugs (anti-psychotics, anti-depressants, antihistamines, and antibiotics) (Haverkamp et al., 2000; Redfern et al., 2003; Fenichel et al., 2004) and many of them (such as cisapride, astemizole, terfenadine, grepafloxacin) have been withdrawn from the market (Farkas and Nattel, 2010; Stockbridge et al., 2013; Ferdinandy et al., 2019).

As a response from the regulatory authorities, the International Council for Harmonisation of Technical Requirements for Pharmaceuticals for Human Use (ICH) guidelines [ICH-S7B, 2005 (Food and Drug Administration, 2005b); ICH-E14, 2005 (Food and Drug Administration, 2005a)] were proposed for rigorous safety testing to avoid similar unacceptable human fatalities (SCD) in association with pharmacological therapy of non-life-threatening pathologies. Unfortunately, no “gold”-standard proarrhythmia screening method exist. Therefore, current safety screening in industry rely on combined use of pre-clinical *in vitro* assessment of HERG function, action potential (AP) and *in vivo* ECG (QT) assays, *in silico* computational (Yang et al., 2016; Ortega et al., 2017; Li et al., 2019) risk prediction [integrated risk assessment (IRA) approach], and clinical ECG studies (Food and Drug Administration, 2005a; Food and Drug Administration, 2005b). Usually, different proarrhythmia markers and score systems [see *Proarrhythmia ECG Markers for Temporal Instability of Repolarization (Used in In Vivo and In Vitro Models*)] are employed to try to increase the predictive value of the overall assessment (Hondeghem et al., 2001; Hondeghem and Hoffmann, 2003; Redfern et al., 2003; Lawrence et al., 2005) with moderate to limited success (Park et al., 2018).

Drug-induced *I*_Kr_/HERG blockade used to be considered the most important factor responsible for proarrhythmia formation. Therefore, for a long time, safety tests largely focused on detection of HERG-blocking potential of test compounds. As a result, this approach led to elimination of potentially promising drug candidates from the developmental pipeline solely on the basis of their potential to block the HERG channel (Carlsson, 2001; Bouder, 2007; Pugsley et al., 2008). HERG blockade plays an inevitably important role in proarrhythmia formation (Yap and Camm, 2003; Hancox et al., 2008); though, HERG block on its own does not necessarily lead to proarrhythmia since simultaneous reduction in other ion currents—such as in *I*_Na_ or *I*_Ca_—that may have anti-arrhythmic effects could modify the overall proarrhythmic potential of the drug (Van Opstal et al., 2001; Belardinelli et al., 2003; Thomsen et al., 2004; Anderson, 2006). On the other hand, compounds without HERG-blocking characteristics may still have proarrhythmic side effect *via* the inhibition of repolarizing ion currents such as the slow delayed rectifier potassium current *I*_Ks_ or the inward rectifier potassium current *I*_K1_ (Pugsley et al., 2008; Roden, 2008; Kannankeril et al., 2010; Pollard et al., 2010). Based on the above, pharmaceutical industry has started the Comprehensive *in vitro* Proarrhythmia Assay (CiPA) initiative to work with validated assays, and to fill gaps in the internal proarrhythmia assessment with the overall goal to comply with the ICH-S7B guidelines. This new approach consists of three main elements: 1) systematic measurement of the effect of drug candidates on multiple human cardiac ion currents (*I*_Kr_, *I*_Ks_, *I*_Na,peak_, *I*_Na,late_, *I*_K1_, *I*_to_, *I*_Ca,L_) in heterologous expression systems (Kramer et al., 2013; Cavero and Holzgrefe, 2014; Colatsky et al., 2016), 2) *in silico* integration of these ion channel effects, and 3) evaluation of the drug effect on integrated biological systems such as on stem-cell-derived cardiomyocytes (Colatsky et al., 2016; Crumb et al., 2016; Huang et al., 2017). According to a recent survey on current industrial safety screening practice (Authier et al., 2017), 90% of the pharmaceutical companies use cell lines expressing cardiac ion channels, 50–60% of them perform ion channel binding assays, cardiac AP recordings or use human induced pluripotent stem cell-derived cardiomyocytes, and only around one-third of them integrate their electrophysiological results into an *in silico* model in order to better predict the drug effect on AP shape and duration. The CiPA initiative is undoubtedly a promising approach; however, its overall implementation is not sufficient yet.

The other major challenge in proarrhythmia screening derives from the huge inter-individual differences in susceptibility to arrhythmia, which makes the overall risk stratification particularly difficult. Gender, sex hormones, K^+^ homeostasis, and—most importantly—certain diseases highly influence the cardiac “repolarization reserve”, defined as the ability of cardiomyocytes to maintain sufficient repolarization despite repolarization-prolonging (mostly K^+^ channel blocking) effects *via* compensatory increase of non-affected “reserve” outward K^+^ currents (Roden, 1998; Varro and Baczko, 2011).

Apart from a decrease in serum K^+^ concentration that results in a massive prolongation of cardiac repolarization, cardiovascular and metabolic diseases such as congestive heart failure (Kjekshus, 1990), cardiac hypertrophy, hypertrophic and dilated cardiomyopathy (Decker et al., 2009), ischemia (Dutta et al., 2016), congenital LQTS (El-Sherif and Turitto, 1999), or diabetes mellitus (Mcnally et al., 1999; Whitsel et al., 2005) play the most important role in decreasing repolarization reserve capacity and thereby in increasing susceptibility to proarrhythmia.

Despite the fact that drug-induced TdP occur mostly in patients with reduced cardiac repolarization reserve, current safety assessments rely still mainly on tests performed on healthy animals with intact repolarization or on their tissues/cells (Food and Drug Administration, 2005a; Food and Drug Administration, 2005b). Consequently, new animal models, (i) with increased sensitivity to channel blockers other than only HERG, such as *I*_Ks_ or *I*_K1_, and (ii) representing different degrees of impairment in their cardiac repolarization reserve were suggested to employ in proarrhythmia research (Hornyik et al., 2020).

Sex hormones also can significantly alter the individual's repolarization reserve capacity and therefore affect susceptibility to arrhythmia. Women are at higher risk for drug-induced prolongation of repolarization and drug-induced TdP (Lehmann et al., 1996; Benton et al., 2000; Wolbrette, 2003; Gowda et al., 2004). This is due to sex hormone effects on cardiac ion currents/channels (Yang and Clancy, 2010; Odening and Koren, 2014): estrogen prolongs cardiac repolarization by decreasing *I*_Ks_ (Drici et al., 1996) and *I*_Kr_ (Kurokawa et al., 2008; Ando et al., 2011) and by increasing *I*_Ca,L_ (Odening et al., 2012a; Odening et al., 2012b) and NCX expression (Chen et al., 2011); therefore, estrogen reduces repolarization reserve and favors drug-induced arrhythmia. In contrast, testosterone and progesterone both increase repolarization reserve by increasing *I*_Ks_ [testosterone (Liu et al., 2003)/progesterone (Furukawa and Kurokawa, 2008)], *I*_K1_ and *I*_Kr_ (testosterone) (Liu et al., 2003), decreasing *I*_Ca,L_ [testosterone (Furukawa and Kurokawa, 2008)/progesterone (Odening et al., 2012a; Odening et al., 2012b)], and upregulating SERCA expression [progesterone (Odening et al., 2012a; Odening et al., 2012b; Moshal et al., 2014)], thereby exerting a protective, “anti-arrhythmic” effect against drug-induced proarrhythmia (Odening et al., 2012a; Odening et al., 2012b). These observations have consequences for proarrhythmia research, as female animal models (or animal models with altered hormonal state) might be particularly sensitive in detecting potential ion channel-blocking properties of drug candidates.

In this review, we first give an overview about currently employed proarrhythmia markers, the importance of the choice of species for proarrhythmia research, and the advantages and disadvantages of currently used *in vivo* proarrhythmia models, followed by a detailed description of novel transgenic LQTS rabbit models. Finally, we provide a brief outlook on possible future directions and novel models in the field.

### Proarrhythmia ECG Markers for Temporal Instability of Repolarization (Used in *In Vivo* and *In Vitro* Models)

Based on the clinical observations that TdP mostly occurred in the setting of prolongation of ventricular repolarization/QT prolongation (Haverkamp et al., 2000; Redfern et al., 2003), preclinical and clinical safety tests have for a long time focused on QT prolongation as a surrogate marker for TdP risk (Food and Drug Administration, 2005b). However, it has been known for some time that prolongation of repolarization does not always equally increase pro-arrhythmic risk since numerous drugs that block *I*_Kr_/HERG and prolong QT rarely cause TdP—while others causing less pronounced QT prolongation carry a significant pro-arrhythmic risk (Zhang et al., 1999; Yang et al., 2001; Roden, 2004; Thomsen et al., 2004; Shah and Hondeghem, 2005). Indeed, the extent of QT prolongation did not predict serious ventricular arrhythmias and/or SCD in different rabbit and dog experimental models (Amos et al., 2001; Thomsen et al., 2004; Shah and Hondeghem, 2005; Lengyel et al., 2007a; Jacobson et al., 2011) or in patients with (Strasberg et al., 1983; Yi et al., 1998; Maron et al., 2001; Hinterseer et al., 2009; Hinterseer et al., 2010) or without (Eisenberg et al., 1995; Kusano et al., 2001; Paltoo et al., 2001) congenital/structural heart disease. Thus, these safety tests can yield either false positive results, halting the development of a promising new drug (Roden, 2004; Hondeghem et al., 2007) or false negative results, causing harm to patients with increased susceptibility to arrhythmia. Therefore, there is an unmet need for (i) improved identification of patients at elevated risk for drug-induced arrhythmia (Kaab et al., 2003) and (ii) novel ECG markers with better predictive value for pro-arrhythmic drug adverse effects.

A number of different electrophysiological parameters have been investigated as non-invasive prognostic markers for TdP and SCD risk evaluation both in animal experiments and in the clinical setting. These novel markers mostly derive from the original work by Hondeghem *et al*., suggesting the assessment of AP triangulation, instability, and dispersion (Hondeghem et al., 2001). These markers were later supplemented by evaluation of disturbances in cardiac wavelength (Hondeghem et al., 2007; Hondeghem, 2016). The importance of an increased spatial (transmural, apico-basal, or inter-ventricular) dispersion of repolarization in reentry-based arrhythmia formation was further supported by other studies (Antzelevitch, 2008; Barbhaiya et al., 2013; Guerard et al., 2014). Therefore, T_peak-end_—the length from the beginning of the T wave (T_p_) to the end (T_e_) on ECG—that reflect the transmural dispersion of repolarization was suggested to use as an indicator of arrhythmic risk, although, no clear consensus about the value of this parameter has been reached so far (Antzelevitch, 2008; Barbhaiya et al., 2013; Meijborg et al., 2014). Of note, dispersion of repolarization was recently identified (experimentally and by computational modeling) as cause for triggered activity/EAD formation, further enhancing the need to assess dynamic dispersion of repolarization noninvasively (Liu et al., 2018).

The temporal instability of cardiac repolarization can be described by different variables characterizing the small beat-to-beat fluctuations in the QT interval as QT variability [for a recent review see (Baumert et al., 2016)]. Beat-to-beat variability of repolarization can be quantified by calculating the short-term variability of the QT (STV_QT_) (Thomsen et al., 2004). First in the chronic AV-block dog model, then in other animal experimental proarrhythmia models (Thomsen et al., 2004; Lengyel et al., 2007a; Jacobson et al., 2011) and in clinical settings (Hinterseer et al., 2008; Hinterseer et al., 2009; Hinterseer et al., 2010; Oosterhoff et al., 2010), it has been repeatedly shown that STV_QT_ has a higher predictive value for proarrhythmia risk than the overall prolongation of repolarization (QT duration). STV_QT_ has been used in several studies to confirm the safety of different drugs (Oros et al., 2006; Antoons et al., 2010; Varkevisser et al., 2012), to characterize pro-arrhythmic drug effects (Thomsen et al., 2004; Thomsen et al., 2006b; Kristof et al., 2012), and to assess temporal repolarization instability in patients with co-morbidities associated with repolarization disturbances (Orosz et al., 2015a; Orosz et al., 2015b; Orosz et al., 2017).

Based on the above, it is justified and required to assess and validate measures of temporal QT variability in animal experimental models of proarrhythmia.

## Current *In Vivo* Models for Pre-Clinical Proarrhythmia Safety Screening and Their Limitations

### Choice of Models: Species Differences in Repolarizing Currents

Cardiac electro-mechanical function shows large inter-species variability. These species differences are especially striking in regard to cardiac repolarization that is governed by tightly regulated activities of various inward and outward ion currents.

**Figure 1:**
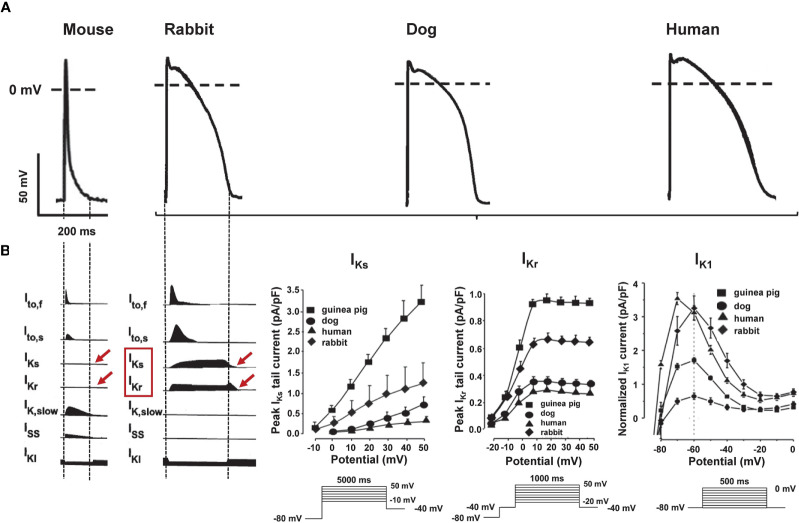
Species differences in repolarizing ion currents. **(A)** Representative action potential (AP) recordings [modified from (Rudy et al., 2008; Blenck et al., 2016)] and **(B)** schematic illustration of the main repolarizing ion currents in mouse, rabbit, dog, and human cardiomyocytes [modified from (Nerbonne et al., 2001)]. Panel **(C)** shows species differences in I_Ks_, I_Kr_, and I_K1_ in rabbit, dog, and human.

Small rodents like mice or rats are commonly used animals for studying ischemia-induced arrhythmias (which are often linked to conduction properties), since cardiac conduction properties in these animals are governed by Na^+^ currents, Ca^2+^ currents, and connexin function that are very similar to those in human (Derangeon et al., 2012; Kaese and Verheule, 2012). Furthermore, they have the advantage of low cost, short life-cycle, and their genetic manipulation is much easier than that in larger animals (see *Genetically Modified Mouse Models With Reduced Repolarization Reserve*). On the other hand; however, they have limited value in studying (prolonged) repolarization-related arrhythmogenesis: in mice and rats the fast and slow transient outward (*I*_to_), and the delayed rectifier (*I*_K,slow1_ and *I*_K,slow2_) (Xu et al., 1999; Brunner et al., 2001; Liu et al., 2008) voltage-gated potassium currents play a major role in repolarization—while in dogs, rabbits, and humans the rapid and slow delayed rectifier potassium currents (*I*_Kr_ and *I*_Ks_) are the major determinants of cardiac repolarization (Varro et al., 1993; Nerbonne, 2000; Nerbonne and Kass, 2005; Grandy et al., 2007; Saito et al., 2009; Yang et al., 2014). The exact role of *I*_Kr_ and *I*_Ks_ in small rodents is still not well known and controversial (Nerbonne and Kass, 2005; Grandy et al., 2007; Saito et al., 2009; Yang et al., 2014). As a result, the shape of their AP is different compared to larger animals and humans (triangular shape vs. rectangular AP with prominent plateau phase) (Nerbonne and Kass, 2005; Grandy et al., 2007; Saito et al., 2009; Yang et al., 2014), as well as their pharmacological responses to proarrhythmic potassium channel blocking drugs (**Figures 1A, B**).

Acute *I*_Kr_-blocker administration, for example, prolongs repolarization in dogs, rabbits, and humans but not in mice or rats (Varro et al., 2000; Nagy et al., 2009; Jost et al., 2013; Yang et al., 2014). Chronic administration of the *I*_Kr_-blocker dofetilide that prolongs repolarization in larger animals and human subjects by blocking HERG channels on the other hand, lengthen repolarization in mice—at least partly—by increasing *I*_Na,L_ (via phosphoinositide 3-kinase pathway) (Yang et al., 2014). Based on the above, mice and rats have serious limitations in drug-induced proarrhythmia research with little translational potential.

In contrast, the function and gating kinetics of various cardiac potassium channels are very similar in dogs, rabbits, and humans with *I*_Kr_ and *I*_Ks_ as main repolarizing ion currents in all three species (Varro et al., 1993; Nerbonne, 2000; Nerbonne and Kass, 2005; Jost et al., 2013), with slight inter-species differences: In humans and dogs—just as in most mammals—*I*_to_ is formed of two distinct subtypes named as *I*_to,fast_ and *I*_to,slow_—with fast and slow recovery from inactivation, determined by Kv3.4 and Kv1.4, respectively (Patel and Campbell, 2005) as opposed to rabbits, where *I*_to,slow_ is the primary transient Kv current in the left ventricle (Fedida and Giles, 1991), while in the right ventricle *I*_to,fast_ and its role in LQT1 related arrhythmogenesis has recently been confirmed (Choi et al., 2018). The repolarization capacity of rabbits and dogs is more robust than that in humans due to higher *I*_K1_, *I*_Kr_, and *I*_Ks_ current densities (Jost et al., 2013; Husti et al., 2015) (**Figure 1C**).

In summary, the rabbit has a prominent role in arrhythmia research, since: (i) the shape of AP (Varro et al., 1993) and the function and gating kinetics of the underlying cardiac ion channels/currents (Nerbonne, 2000), (ii) the myocardial mechanical function (Jung et al., 2012), (iii) the relative effective heart size relating cardiac mass to the frequency of VF (Panfilov, 2006), and (iv) their responses to pharmacological interventions (Harken et al., 1981) show very close resemblance to human cardiac physiology. Based on the above-described species differences in various ion currents, the rabbit could have advantages over dog models when testing the proarrhythmic potential of drugs with *I*_Kr_, *I*_Ks_, or *I*_K1_-blocking properties since they show very good similarity to human physiology in these currents and are cheaper, easier to handle, and to breed than dogs and can be modified genetically. Dogs or guinea pigs, on the other hand, may be better suited to use for studying arrhythmogenesis in which *I*_to_-inhibition plays an important role.

### *In Vivo* Proarrhythmia Models With Reduced Repolarization Reserve

Both drug-induced and genetically modified animal models with impaired repolarization reserve have been generated in various species (such as dogs, mice, and rabbits) and utilized to investigate drug-induced proarrhythmia and its underlying mechanisms [reviewed in (Salama and London, 2007; Lang et al., 2016)]. In addition, animal models with cardiac diseases—e.g. chronic heart failure, hypertrophic cardiomyopathy, diabetes mellitus etc.—leading to electrical remodeling-associated alterations of repolarization reserve can be used for proarrhythmia research. These models have the advantage that they mimic some of the diseases that also predispose human patients to drug-induced arrhythmias.

One main shortcoming of drug-induced animal models, however, is the fact that drugs have to be administered continuously to sustain the drug-induced reduction of repolarization reserve, thus impeding detailed investigation of (long-term) proarrhythmia in free-moving, non-anesthetized animals.

One main short-coming of genetically modified mouse models—the only genetic models with reduced repolarization reserve available until 2008—are the pronounced species differences in cardiac electrophysiology with different ion currents conveying cardiac repolarization in human and murine cardiomyocytes (Varro et al., 1993; Nerbonne, 2000; Nerbonne and Kass, 2005; Jost et al., 2013) as highlighted in the subchapter above.

High cost and need for special technical expertise for example are considered as main shortcomings for the generation of disease-related animal models with cardiac electrical remodeling.

#### Drug-Induced Animal Models With Reduced Repolarization Reserve

One frequently used drug-induced model is the methoxamine-sensitized rabbit model: anesthetized rabbits are sensitized with the selective α-adrenoceptor-agonist methoxamine, which makes them particularly prone to develop drug-induced ventricular tachycardia when exposed to HERG/*I*_Kr_-blocking drugs (Carlsson, 2008; Carlsson et al., 2009). The choice of the anesthetic regimen, however, strongly influences the extent of proarrhythmia development in this model—as the different anesthetics all have different intrinsic cardiac ion channel-blocking properties (Carlsson, 2008; Inaba et al., 2011), indicating the importance of the degree of ion channel blockade in these “mixed” drug-induced models. Pro- and anti-arrhythmic properties of various different drugs have been tested and classified as having a low, intermediate, or high pro-arrhythmic potential for drug-induced TdP using this model (Diness et al., 2008; Carlsson et al., 2009; Jacobson et al., 2011; Mow et al., 2015; Varkevisser et al., 2015). However, as the model depends on α-adrenoceptor sensitization, the full extent of pro-arrhythmic potential of drugs that concomitantly block *I*_Kr_ and α-adrenoceptors (such as quinidine, cisapride, quinolone) may not be fully appreciated by this model (Carlsson, 2008; Mow et al., 2015).

**Figure 2:**
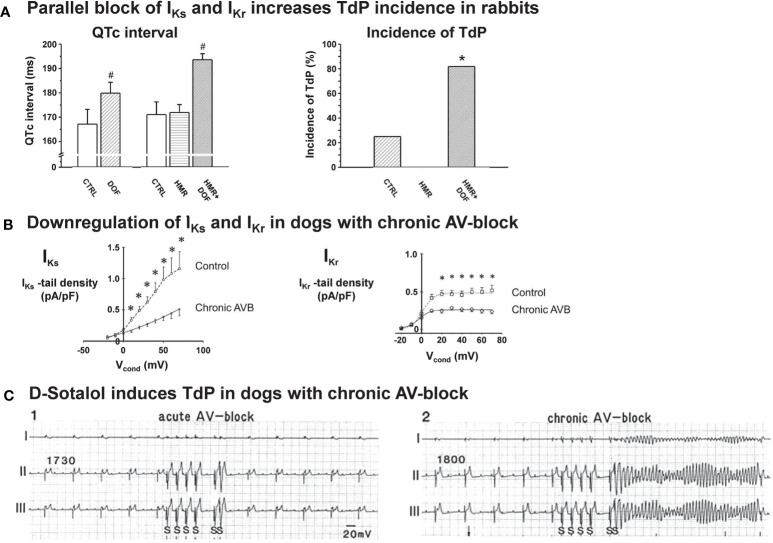
Pro-arrhythmia detection with acquired LQT rabbit and chronic AV-block dog models. **(A)** Left panel: The QTc interval was significantly prolonged by the I_Kr_ blocker dofetilide and was further prolonged by the combination of dofetilide and the I_Kr_ blocker HMR1556 given in any order, while I_Ks_ block alone did not prolong the QTc in anesthetized rabbits. Right panel: I_Kr_ block caused TdP in some of the animals, I_Ks_ block alone did not induce TdP in anesthetized rabbits. Importantly, combined acute pharmacological block of I_Ks_ and I_Kr_, given in any order, caused a significant increase in TdP incidence. #p < 0.05 vs. baseline control in the same group; *p < 0.05 vs. dofetilide. N=7-11 animals/group. CTRL: Control, DOF: Dofetilide, HMR: HMR-1556. Modified from (Lengyel et al., 2007a). **(B)** In right ventricular cardiomyocytes isolated from dogs with chronic AV-block and marked myocardial hypertrophy, I_Ks_ tail current density was reduced by approximately 55% (n=9), while I_Kr_ tail current density was reduced by 45% (n=14) compared to those isolated from control animals. *p < 0.05 vs. control myocytes. Modified from (Volders et al., 1999). **(C)** Standard bipolar ECG lead recordings illustrating a marked difference in the arrhythmogenic response to administration of the selective I_Kr_ blocker D-sotalol in dogs with acute (left panel) and chronic AV-block (right panel). The pacing protocol involved a basic pacing cycle length (CL) of 1730 ms in acute AV block, and 1800 ms spontaneous idioventricular CL in chronic AV-block. Arrhythmia provocation included a short/long/short sequence at 600 ms CL for 4 beats followed by 2 paced beats with 1200 and 350 ms cycle lengths. No TdP was observed in acute AV-block dogs, while 60% of animals with chronic AV-block exhibited TdP. Modified from (Vos et al., 1998).

Other frequently employed drug-induced rabbit models are based on the *ex vivo* blockade of HERG/*I*_Kr_ by E-4031 (Choi et al., 2002; Maruyama et al., 2011; Parikh et al., 2012; Lau et al., 2015) or by dofetilide (Farkas et al., 2006; Dhein et al., 2008; Orosz et al., 2014), thus generating a drug-induced rabbit model representing LQT2 characteristics. Similar to the genetic reduction of *I*_Kr_ in congenital LQT2, the drug-blockade of *I*_Kr_ by E-4031 or dofetilide leads to a high propensity for arrhythmia development in *ex vivo* Langendorff-perfused rabbit hearts—particularly when perfused with low K^+^ and Mg^2+^ solutions (Maruyama et al., 2011; Milberg et al., 2011). Similarly, QTc prolongation and arrhythmia susceptibility is particularly high when HERG/*I*_Kr_ blockade is combined with KvLQT1/*I*_Ks_ blockade by HMR-1556 (Lengyel et al., 2007a) (**Figure 2A**).

#### Genetically Modified Mouse Models With Reduced Repolarization Reserve

Despite apparent differences between human and mouse electrophysiology—e.g., different ion currents conveying cardiac repolarization in human and murine cardiomyocytes, a different shape of the AP, and a magnitude-faster heart rate in mice [reviewed in (Nerbonne, 2000); (Salama and London, 2007); (Baczko et al., 2016)], the first transgenic and knock-out animal models of LQTS were mouse models (London et al., 1998). The reason for this was that genetic manipulation has for a long time nearly exclusively been feasible in mice and not in other larger mammals such as rabbits. Transgenic models based on mutations in human potassium channel genes or knock-out/knock-in models of mouse potassium channel genes could only partially mimic the characteristics of human patients with impaired repolarization reserve [reviewed in detail in (Nerbonne et al., 2001); (Lang et al., 2016); (Ziupa et al., 2019)], while genetically modified mouse models with mutations in the sodium channel gene (SCN5A; LQT3) more closely mimic the human long QT disease phenotype with AP duration (APD) and QT prolongation as well as spontaneous life-threatening ventricular arrhythmia (Nuyens et al., 2001; Fabritz et al., 2003), since *SCN5A* drives the majority of depolarizing Na^+^ currents in both human and murine cardiomyocytes (Derangeon et al., 2012).

None of these mouse models, however, have been systematically used for proarrhythmia research—mainly due to the fact that *I*_Kr_ current (the most frequent target for drugs inducing proarrhythmia) plays no major role in cardiac repolarization in mice.

#### Animal Models With Structural/Electrical Remodeling and Reduced Repolarization Reserve

Several experimental animal models of different species exist that are characterized by impaired repolarization reserve associated with cardiac structural and/or electrical remodeling. The most thoroughly characterized of these is the canine cardiac volume overload model with chronic atrioventricular block (Vos et al., 1995). Three months following AV-node ablation, these dogs exhibit severe bradycardia and eccentric, biventricular myocardial hypertrophy without heart failure (Vos et al., 1998). Importantly, the model is characterized by heterogeneous prolongation of APD at baseline and further APD-prolongation and early afterdepolarization (EAD) development upon administration of *I*_Kr_-blocker *d*-sotalol, indicating increased susceptibility to drug-induced TdP (Vos et al., 1998) (**Figure 2C**). A significant downregulation of potassium currents, including *I*_Ks_—a key player in ventricular repolarization reserve in mammals including humans (Varro et al., 2000; Volders et al., 2003; Jost et al., 2005)—was observed in both left and right ventricular cardiomyocytes isolated from dogs with chronic AV block (Volders et al., 1999) (**Figure 2B**). In addition, *I*_Kr_ density was reduced by 45% in right ventricular myocytes, while left ventricular (LV) *I*_Kr_ density was unchanged (Volders et al., 1999).

The ability of the model to detect drug-induced TdP was validated (Takahara et al., 2006) with several drugs such as the H_1_ antihistamine terfenadine (Monahan et al., 1990), the antipsychotic drug sertindole (Thomsen et al., 2003), and the D_2_ dopamine receptor antagonist sulpiride (Sugiyama et al., 2002). Moreover, with this model, it was demonstrated for the antibiotics moxifloxacin and azithromycin that QT prolongation does not necessarily cause TdP (Thomsen et al., 2006a). An important advantage of this model is an improved proarrhythmia reproducibility within the same experimental animal (Verduyn et al., 2001). There are, however, some disadvantages that may prevent this model from becoming widely used for proarrhythmia research: it is a relatively expensive, time consuming (at least 3 months have to pass before ventricular hypertrophy and increased susceptibility to arrhythmia develops) and low throughput method that requires special technical expertise for performing AV-node ablation (Vos et al., 1998).

In diabetes, a moderate remodeling-associated QT prolongation has been shown (Giunti et al., 2007), and diabetes has been associated with increased risk of SCD (Gill et al., 2009), indicating the patients' higher susceptibility to proarrhythmia. Similarly, in rabbit and dog models of diabetes mellitus, a mild prolongation of repolarization and a decreased repolarization reserve due to downregulation of *I*_Ks_ and *I*_to_ (reversible by insulin treatment) were observed (Lengyel et al., 2007b; Lengyel et al., 2008). The authors are not aware, however, of any published studies using experimental diabetes animal models in species with repolarization relevant to human (i.e. not mice or rats) for testing pro-arrhythmic effects of drugs in this “patient” cohort.

## Transgenic LQTS Rabbit Models With Impaired Repolarization Reserve (LQTS)

Since cardiac electrophysiological characteristics of the rabbit is much closer to humans than that of mice or rats (Varro et al., 1993; Nerbonne, 2000; Nerbonne and Kass, 2005; Jost et al., 2013),—similar potassium currents (mainly I_Kr_ and I_Ks_) convey the cardiac repolarization in rabbits and humans—transgenic LQTS rabbit models were generated as soon as it became technically feasible (Bosze et al., 2016) to better mimic pathophysiology of (human) LQTS patients with decreased repolarization reserve, who are most vulnerable to the development of drug-induced arrhythmias.

### Generation of Transgenic LQTS Rabbit Models

To generate transgenic LQTS rabbit models with impaired repolarization reserve, the so-called “dominant-negative” transgenic strategy, which describes the fact that the co-assembly of mutated and normal channel subunits completely disrupts the overall ion channel function, was utilized to decrease the expression of functionally normal repolarizing potassium channel proteins. All available transgenic LQTS rabbit models have been engineered by beta-myosin heavy chain promoter-driven cardio-selective over-expression of mutated human genes encoding for voltage-gated K^+^ channels such as *KCNQ1*/KvLQT1 (KvLQT1-Y315S, LQT1)*, KCNH2*/HERG (HERG-G628S, LQT2), or *KCNE1*/minK (KCNE1-G52R) (Brunner et al., 2008; Major et al., 2016).

To generate the transgenic founder animals, the pronuclear microinjection technique was used. Superovulation was induced in wild-type (WT) rabbits using hormonal stimulation with FSH and GnRH-analogues, and inseminated oocytes were microinjected with transgenic mutant DNA-constructs and re-implanted into foster mothers (Brunner et al., 2008; Major et al., 2016). Mating of the resulting transgenic F0 founders with female WT rabbits resulted in vertical transmission with 50% transgenic and 50% WT offspring. To generate double-transgenic LQT2–5 rabbits, LQT2 male and LQT5 female rabbits were cross-bred (Hornyik et al., 2020).

### Electrophysiological Characteristics and Arrhythmogenic Mechanisms in the Transgenic LQTS Models

**Figure 3:**
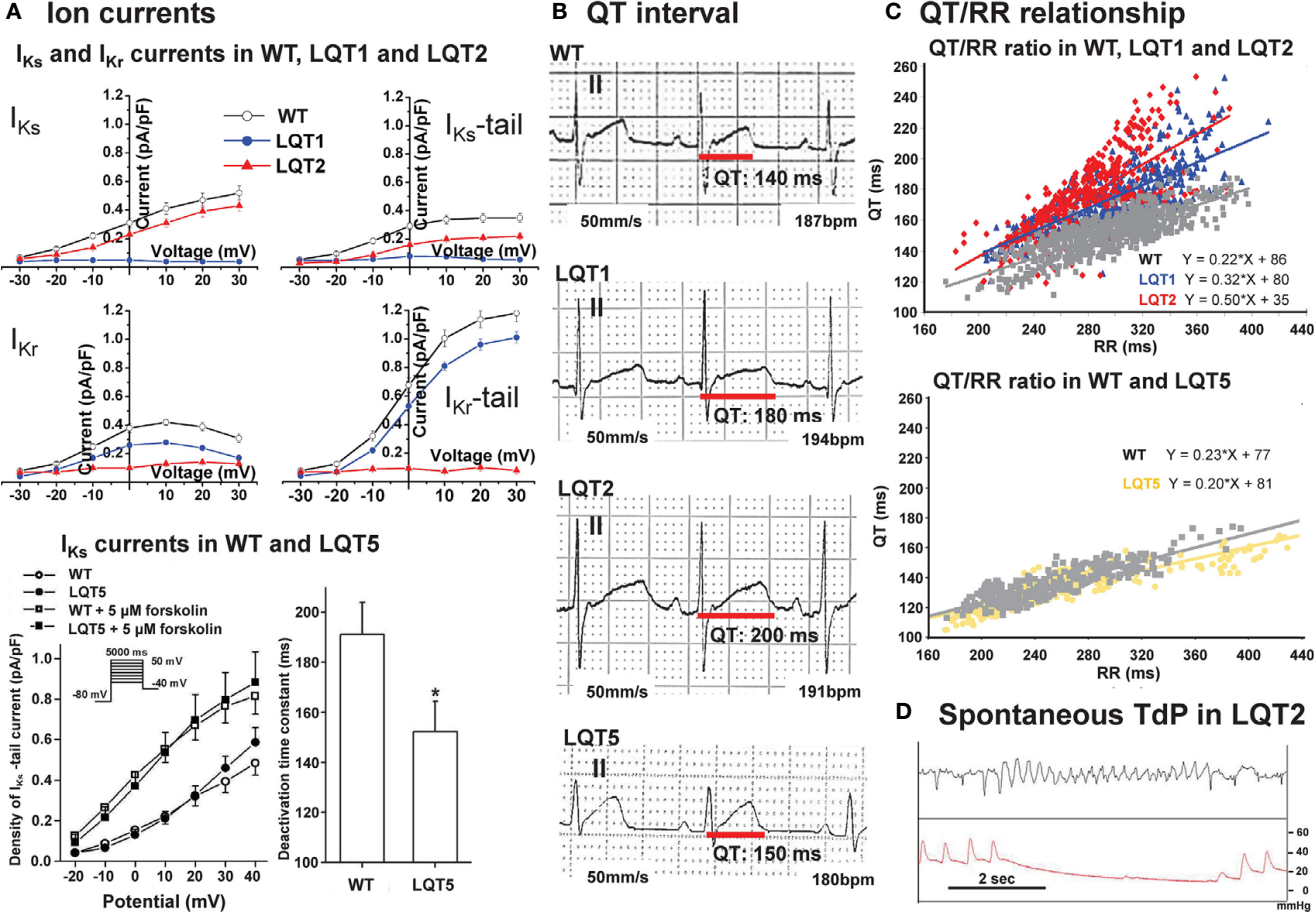
Baseline electrical characteristics of transgenic LQTS rabbits. **(A)** Upper panel: IV-curves of I_Ks_ steady (left)/tail (right) and I_Kr_ steady (left)/tail (right) in cardiomyocytes isolated from wild-type (WT), LQT1, and LQT2 rabbit hearts, indicating loss of I_Ks_ in LQT1 and loss of I_Kr_ in LQT2 [modified from (Brunner et al., 2008)]. Lower panel: IV-curves of I_Ks_ in absence and presence of 5 µM forskolin in WT or transgenic LQT5 rabbit ventricular myocytes. Bar diagrams illustrate a reduced deactivation time constant in transgenic LQT5 ventricular myocytes [modified from (Major et al., 2016)]. **(B)** Representative ECG tracings indicating differences in QT interval in WT, LQT1, LQT2, and LQT5 rabbits [ECG from WT, LQT1 and LQT2 modified from (Brunner et al., 2008)]. **(C)** QT/RR relationship assessed with telemetric ECG in free moving rabbits: WT, LQT1, and LQT2 in upper panel [modified from (Brunner et al., 2008)], in lower panel in WT and LQT5 [modified from (Hornyik et al., 2020)]. **(D)** ECG and blood pressure tracing of LQT2 rabbit with spontaneous ventricular torsade-de-pointes (TdP) tachycardia [modified from (Brunner et al., 2008)]. *p < 0.05 vs. WT.

In LQT1 or LQT2 rabbit cardiomyocytes the repolarizing potassium currents *I*_Ks_ (LQT1) or *I*_Kr_ (LQT2), respectively, are completely eliminated. This results in a prolongation of APD on the cellular and whole heart levels and a prolongation of ventricular refractoriness and QT interval duration *in vivo* (Brunner et al., 2008; Odening et al., 2010). This prolongation of APD/QT is particularly pronounced at slower heart rates, leading to a steeper QT/RR ratio—particularly in LQT2 (Brunner et al., 2008) (**Figures 3A–C**). Moreover, these models demonstrate an increased temporal instability of QT duration with an increased STV_QT_ (Hornyik et al., 2020) and spontaneous polymorphic VT in LQT2 [158] (**Figure 3D**).

In transgenic LQT5 rabbit cardiomyocytes, in contrast, the biophysical properties of *I_Ks_* and *I_Kr_* are altered with accelerated deactivation kinetics (Major et al., 2016)—but overall *I_Ks_* and *I_Kr_* current densities are not reduced. Consequently, these rabbits exhibit only a partial phenotype with no significant prolongation of whole heart APD (Major et al., 2016; Hornyik et al., 2020), and only a very slightly prolonged QT interval at baseline without changes in QT/RR ratio (Major et al., 2016; Hornyik et al., 2020) (**Figures 3A–C**), but exhibit an increased short-term beat-to-beat variability of the QT (Major et al., 2016). Due to their reduced repolarization reserve, the phenotype can be augmented by the *I*_Kr_-blocking drug dofetilide, which further increased short-term variability of QT and promoted drug-induced VT (Major et al., 2016).

**Figure 4:**
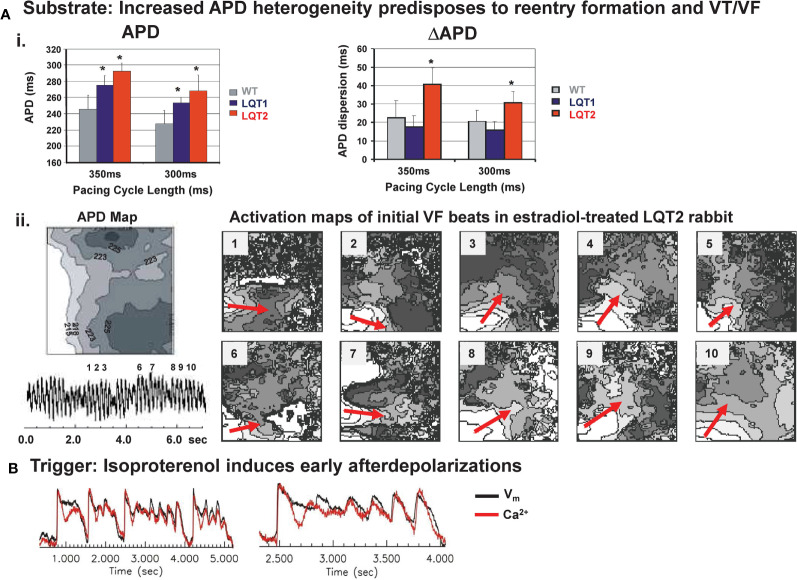
Mechanisms of arrhythmogenesis in transgenic LQT2 rabbits. **(A)** Upper panel (i): Bar graphs of action potential durations (APD) and dispersion of APD (ΔAPD) in Langendorff-perfused hearts of WT, LQT1, and LQT2 rabbits indicate longer APD and increased APD dispersion in LQT2 hearts, *p < 0.05 [modified from (Brunner et al., 2008)]. Lower panel (ii): optical mapping APD map (left; indicated are isochrones of APD, in ms) and activation maps (right) of initial beats of ventricular tachycardia/fibrillation (VF) in an estradiol-treated LQT2 heart indicate reentry formation (indicated by red arrows) in line with the regional APD heterogeneities [modified from (Odening et al., 2012b)]. **(B)** Representative trace of Ca^2+^ oscillations and early afterdepolariaztion (EAD) in estradiol-treated LQT2 rabbit heart after AV-node ablation and bolus application of isoproterenol (140 nM). Black line indicates changes in voltage fluorescence signal (Vm); red line indicates changes in Ca^2+^ signal [modified from (Odening et al., 2012b)].

Studies in transgenic LQT1 and LQT2 rabbits highlight the major role of an enhanced (spatial and temporal) dispersion of repolarization in LQTS-related arrhythmogenesis: in LQT2 rabbit hearts, an increased spatial dispersion of APD was observed throughout left and right ventricles (Brunner et al., 2008; Odening et al., 2013) (**Figure 4A**). Dispersion of repolarization can also occur in a dynamic spatio-temporal fashion with pronounced beat-to-beat alternations and “out-of-phase” heterogeneities between adjacent regions, the so-called “discordant alternans”. In transgenic LQT2 rabbit hearts, this discordant alternans developed at physiological heart rates and preceded VT/VF formation (Ziv et al., 2009). VT/VF were easily inducible with LV epicardial stimulation (Brunner et al., 2008), and, importantly, LQT2 rabbits even developed spontaneous polymorphic VT and SCD (Brunner et al., 2008; Odening et al., 2012b), thus representing the first transgenic animal models mimicking the complete electrical phenotype of LQT2. Transgenic LQT1 rabbits with a more homogeneously prolonged APD without substantial dispersion of repolarization within the LV at physiological/normal heart rates, in contrast, developed no spontaneous VT or SCD (Brunner et al., 2008). When LQT1 hearts were further stressed, however, by continuous tachypacing or AV-ablation to induce cardiac tachymyopathy (Lau et al., 2015), or complete AV-block (Kim et al., 2015), respectively, APD dispersion increased, spatially discordant alternans developed and VT/VF was easily inducible or occurred spontaneously. Transgenic LQT5 rabbits demonstrated an increased apico-basal APD heterogeneity compared to healthy WT hearts at baseline—despite their overall “normal” APD (Hornyik et al., 2020).

In addition to increased temporal instability and regional heterogeneity of repolarization that form the electrical “substrate” that facilitates re-entry formation, an increased sympathetic nervous system activity serves as “trigger” for EADs in long QT-related arrhythmogenesis (Antzelevitch, 2007; Brunner et al., 2008; Ziv et al., 2009). In LQT2 cardiomyocytes, beta-adrenergic stimulation related EADs developed during sudden sympathetic surge (**Figure 4B**), while continuous perfusion with beta-agonist isoproterenol prevented EAD formation (Brunner et al., 2008; Liu et al., 2012; Odening et al., 2012b). In LQT1 cardiomyocytes, in contrast, continuous beta-adrenergic stimulation facilitated the occurrence of EADs (Liu et al., 2012). Different time courses in sympathetic activation of cardiac ion currents may explain why different sympathetic modes (sudden surge vs. continuous activation) are associated with arrhythmia formation in different genotypes of LQTS: upon sympathetic stimulation, beta-1 receptor mediated activation of *I*_Ca,L_—that may elicit EADs—is faster than the activation of *I*_Ks_—that shortens APD in LQT2 and acts as anti-arrhythmic mechanism upon continuous adrenergic stimulation in LQT2. In addition, different modes of arrhythmia initiation and maintenance in different LQTS genotypes were identified. While in LQT2, reentry formation played an important role (Brunner et al., 2008), in LQT1 hearts, a novel mechanistic concept of LQTS-related arrhythmogenesis was identified: arrhythmia was initiated by focal excitations arising particularly from the RV and was maintained by multiple shifting excitation foci and bi-excitability (Kim et al., 2015).

Similarly, as in the LQT5 rabbit models, the phenotype and proarrhythmia could be augmented by ion channel blocking drugs and endogenous factors (as highlighted in the following subchapters).

### Pro-Arrhythmic Effects of Endogenous Factors (Hormones and Metabolites) in Transgenic LQTS Rabbit Models With Impaired Repolarization Reserve

Pronounced sex differences in arrhythmic risk have been identified in patients with congenital and acquired, drug-induced LQTS with a higher risk for cardiac arrhythmic events in women after puberty than men (Lehmann et al., 1996; Locati et al., 1998; Benton et al., 2000; Wolbrette, 2003; Gowda et al., 2004; Yang and Clancy, 2011). Moreover, while the risk for long QT-related arrhythmia is reduced during pregnancy (Seth et al., 2007), it is particularly high risk during the postpartum (particularly in LQT2 patients) (Sauer et al., 2007). In addition, more pronounced QT-prolongation and arrhythmias are observed during luteal than follicular phases of the menstrual cycle (Rodriguez et al., 2001), strongly suggesting that changing sex hormone levels may affect LQTS-related arrhythmogenesis. This has consequences for proarrhythmia research, as female animal models might be particularly sensitive in detecting potential ion channel-blocking properties of drug candidates.

In transgenic LQT2 rabbits, spontaneous ventricular arrhythmia and SCD also often occurred during postpartum (Brunner et al., 2008; Odening et al., 2012b), suggesting the existence of similar arrhythmia-triggering mechanisms as in human LQTS patients. In these models, estradiol exerted a pro-arrhythmic effect with an increased incidence of lethal polymorphic TdP due to changes in APD dispersion and increased EAD formation upon pro-arrhythmic sympathetic stimuli, while progesterone had an anti-arrhythmic, protective effect that was based on a shortening of cardiac refractoriness, a reduced formation of EAD, and stabilizing Ca^2+^ effects (decreased *I*_Ca,L_ density, increased SERCA expression) (Odening et al., 2012b). These studies suggest that progesterone-based therapies may be considered as novel anti-arrhythmic approaches in female LQTS patients; and might be considered as therapeutic add-on in cases of severe drug-induced long-QT related proarrhythmia (Odening et al., 2012b). As estradiol-treated hearts are particularly sensitive to proarrhythmia, their use in proarrhythmia research, in contrast, might increase sensitivity to identify candidates with a pro-arrhythmic potential.

Similarly, the postpartum-related hormones oxytocin and prolactin decreased *I*_Ks_ current densities, thereby prolonging the APD/QT further and predisposing the heart to arrhythmias (Odening et al., 2019).

Hormones as well as other endogenous factors such as certain metabolites may impact on repolarization reserve: it has been demonstrated that (genetic) metabolic disturbances such as propionic acidemia can also cause acquired LQTS (Kakavand et al., 2006; Baumgartner et al., 2007; Jameson and Walter, 2008), thus rendering patients at increased risk for additional drug-induced proarrhythmia. Using rabbit models of LQTS, we could identify a propionic acid-induced reduction of *I*_Ks_ current densities as underlying mechanism (Bodi et al., 2016).

### Transgenic LQTS Rabbit Models for Better Detection of Drug-Induced Ventricular Arrhythmias

Drug-induced proarrhythmia is based on regionally heterogeneous prolongation of cardiac repolarization caused by various groups of drugs blocking multiple ion channels. As it most-often occurs in patients with reduced repolarization reserve (see above), the use of various transgenic LQTS rabbit models (Brunner et al., 2008; Major et al., 2016) with increased sensitivities to potassium channel blocking effects and different degrees of impairment in their cardiac repolarization reserve, is expected to provide more reliable, and more thorough detection of (multi-channel-based) drug-induced ventricular arrhythmias.

Indeed, it has been shown that LQT1 rabbits lacking *I*_Ks_ and LQT5 rabbits with impaired *I*_Ks_ function were particularly sensitive in identifying *I*_Kr_-blocking properties of drugs; while transgenic LQT2 rabbits lacking I_Kr_ demonstrated a particularly high sensitivity to *I*_Ks_*-* or *I*_K1_-blocking drugs (Volders et al., 2003; Jost et al., 2005; Odening et al., 2010; Odening et al., 2013; Hornyik et al., 2020) as further outlined in the following subchapters.

#### Investigation of Drug-Induced Changes in Proarrhythmia Markers in Transgenic LQTS Rabbit Models

Drug-induced arrhythmia is a relatively rare event, and arrhythmia development as “hard endpoint” cannot be directly studied—especially during “first-in-human” clinical trials. Therefore, model systems with increased susceptibility for proarrhythmia are employed for safety testing and changes in various proarrhythmia markers are monitored to assess the pro-arrhythmic potential of the investigated/applied drug.

Several biomarkers have been suggested to use for proarrhythmia screening that reflect changes in various aspects of repolarization such as: (i) duration (QTc, APD), (ii) spatial (T_peak-end_, APD dispersion), and (iii) temporal dispersion (STV_QT_, QT/RR steepness, APD restitution) of repolarization (for more details, see previous sections). These were also employed in the different transgenic LQTS rabbit models.

**Figure 5:**
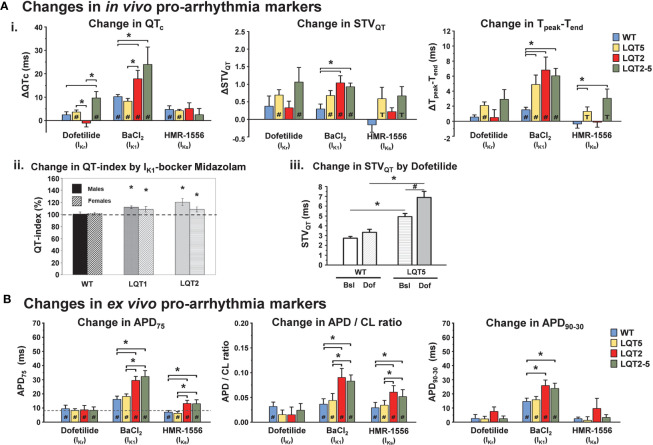
Pro-arrhythmic drug-effects in transgenic LQTS rabbit models: changes in pro-arrhythmia markers. **(A)** Changes in *in vivo* pro-arrhythmia markers. (i.) Bar graphs show changes in QTc, STV_QT_, and T_peak_-T_end_ in anaesthetized animals after i.m. injection of I_Kr_, I_K1_, and I_Ks_-blockers dofetilide, BaCl_2_, HMR-1556, respectively in WT, LQT2, LQT5, and LQT2–5 rabbits [modified from (Hornyik et al., 2020)]. *p < 0.05 inter-genotype comparison, ^#^p < 0.05 vs. baseline, T trend p < 0.1 vs. baseline. (ii.) I_K1_-blocker midazolam-induced change in heart-rate corrected QT-index in free-moving male (solid bars) and female (hatched bars) WT, LQT1, and LQT2 rabbits is also shown. The dashed line represents the mean QT indexes in free-moving rabbits obtained with the genotype-specific correction formula (=100%) [modified from (Odening et al., 2008)]. *p < 0.05 vs. free-moving rabbits of the same genotype. (iii.) Bar graphs indicate I_Kr_-blocker dofetilide-induced increase in STV_QT_ in WT and LQT5 animals [modified from (Major et al., 2016)]. Bsl, baseline; Dof, dofetilide.*p < 0.05 inter-genotype comparison, ^#^p < 0.05 vs. baseline. **(B)** Changes in *ex vivo* pro-arrhythmia markers. Bar graphs of changes in action potential duration (ΔAPD75), action potential duration/stimulation cycle length ratio (ΔAPD/CL ratio and action potential triangulation (ΔAPD90-30) after 10 min perfusion with I_Kr_, I_K1_ and I_Ks_-blockers dofetilide, BaCl_2_ and HMR-1556, respectively in WT, LQT2, LQT5, and LQT2–5 rabbits. *p < 0.05 inter-genotype comparison, ^#^p < 0.05 vs. baseline [modified from (Hornyik et al., 2020)].

Transgenic LQT2 rabbits demonstrated a high sensitivity to *I*_Ks_*-* or *I*_K1_-blocking anesthetic agents such as isoflurane (*I*_Ks_) or the anxiolytic sedative midazolam (*I*_K1_) as demonstrated by a particularly pronounced prolongation of QT and heart-rate corrected QT index as compared to healthy rabbits (Odening et al., 2008) (**Figure 5A**). In addition, a pronounced increase in *in vivo* ECG proarrhythmia markers (QT, STV_QT_, and T_peak-end_) and *ex vivo* monophasic AP related proarrhythmia markers (APD, AP triangulation and APD restitution steepness) indicated higher susceptibility also to other *I*_K1_ and *I*_Ks_ blocking compounds (BaCl_2_ and HMR-1556) in transgenic LQT2 and double-transgenic LQT2–5 rabbits (Hornyik et al., 2020) (**Figures 5A, B**).

LQT1 and LQT5 models; on the other hand, were especially sensitive to *I*_Kr_ blockade (dofetilide, E4031, and erythromycin), as demonstrated *in vivo* by a pronounced increase in QTc, STV_QT_, and T_peak-end_ and *ex vivo* by a prolongation of APD and (in LQT1) APD dispersion (Odening et al., 2010; Ziupa et al., 2014; Major et al., 2016).

#### Investigation of *Ex Vivo* Susceptibility to Arrhythmia in Isolated Transgenic LQTS Rabbit Hearts

**Figure 6:**
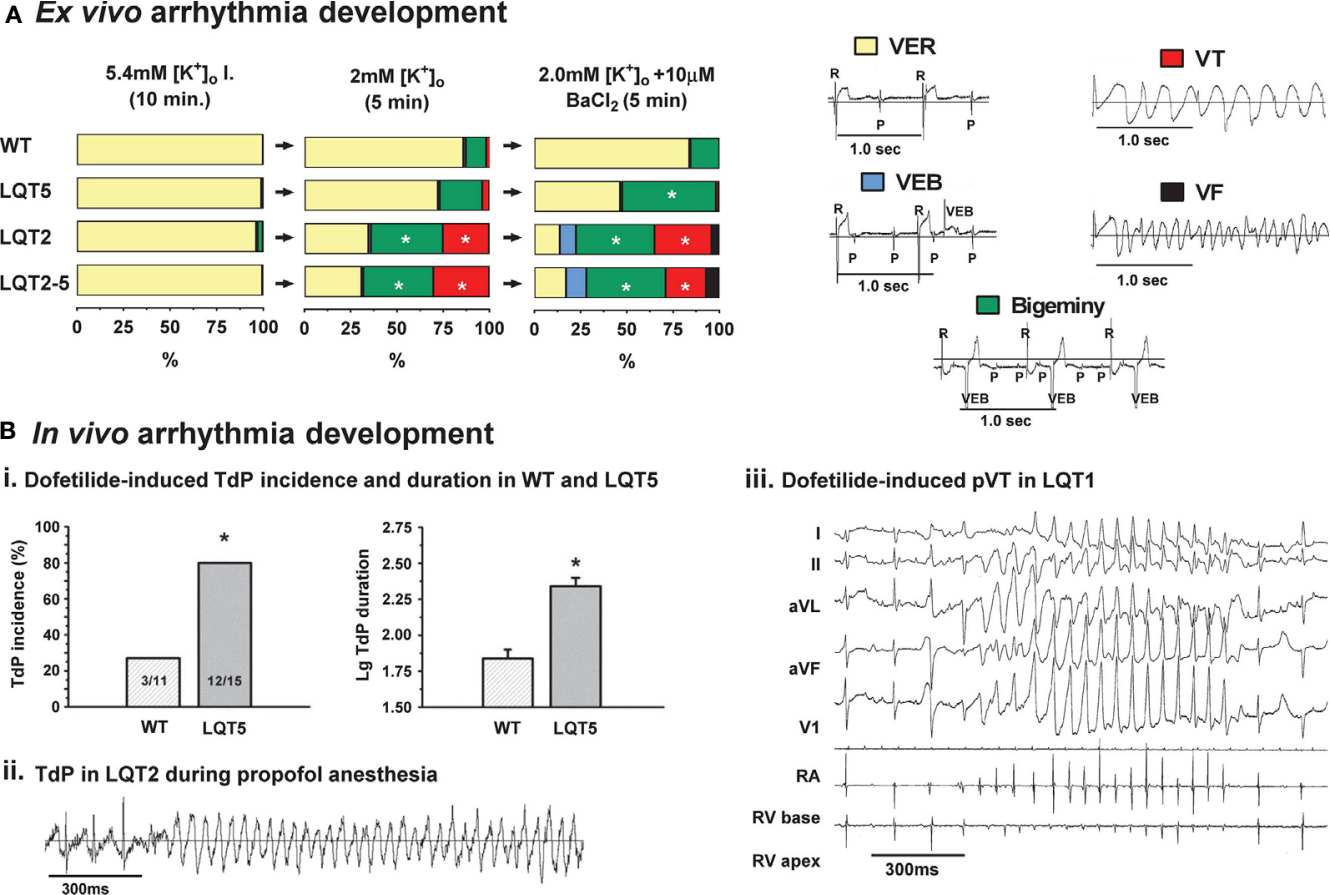
Pro-arrhythmic drug-effects in transgenic LQTS rabbit models: arrhythmia development. **(A)** *Ex vivo* arrhythmia development. Graphs indicating the duration (% of perfusion time) of arrhythmias provoked by perfusing the hearts with low [K^+^]_o_ (2 mM) KH, or with combined low [K^+^]_o_ (2 mM) KH and I_K1_-blocker BaCl_2_ (10 µM) in WT, LQT5, LQT2 and LQT2–5 animals. Inlets show representative ECG recordings of ventricular escape rhythm (VER), ventricular extra beats (VEB), bigeminy, ventricular tachycardia (VT) and ventricular fibrillation (VF) [modified from (Hornyik et al., 2020)]. *p < 0.05 inter-genotype comparison. **(B)** *In vivo* arrhythmia development. (i.) Bar graphs of dofetilide-induced incidence (in %) and log duration of torsade de pointes (TdP) tachycardia in WT and LQT5 rabbits [modified from (Major et al., 2016)]. *p < 0.05 inter-genotype comparison. (ii.) Episode of polymorphic TdP tachycardia in female LQT2 rabbit during propofol anesthesia (acquired with telemetric ECG) [modified from (Odening et al., 2008)]. (iii.) Episode of dofetilide-induced pVT in male LQT1 rabbit during episode of alternating AV 2:1/3:1 block [modified from (Odening et al., 2010)].

The direct assessment of *ex vivo* arrhythmia development in isolated Langendorff-perfused transgenic LQTS rabbit hearts were first performed by Hornyik et al., using different provocation factors—such as bradycardia, low [K^+^]_o_, and/or application of K^+^-channel blockers—that are also crucial in drug-induced proarrhythmia formation in the clinical setting (Hornyik et al., 2020). It was shown that the application of *I*_K1_-blocker BaCl_2_ alone already significantly increased the incidence and duration of ventricular extra beats in LQT2 and LQT2–5 hearts (**Figure 6A**). When the pre-existing temporal, beat-to-beat-QT-instability, and the prolonged, regionally heterogeneous repolarization were even further aggravated by lowering the [K^+^]_o_, the sensitivity for re-entry formation was increased, leading to significantly longer duration and higher incidence of more malignant type of ventricular arrhythmias (VT and VF) in LQT2 and LQT2–5 but not in healthy WT hearts (**Figure 6A**). In LQT5 hearts with less pronounced reduction of repolarization reserve, only less severe types of ventricular arrhythmias (bigeminy) were detected. These observations of a higher sensitivity of transgenic LQTS hearts to potassium channel blocking drugs than normal WT hearts suggests that the use of different transgenic LQTS rabbits might help in more reliably detect drug-induced proarrhythmia.

#### Investigation of *In Vivo* Susceptibility to Arrhythmia of Transgenic LQTS Rabbits

High *in vivo* susceptibility to arrhythmia of transgenic LQTS rabbit models were demonstrated as early as the first characterizations of the models were performed.

In transgenic LQT2 rabbit models, exhibiting regionally heterogeneous prolongation of repolarization, VT and VF easily occurred spontaneously resulting in high SCD rates (Brunner et al., 2008). In addition, a further reduction of repolarization reserve by the *I*_Ks_-blocker isoflurane or by the *I*_to_-/*I*_Ks_-blocker propofol led to increased proarrhythmia with a variety of arrhythmic events such as AV 2:1 blocks, T-wave alternans, PVCs, bigeminy, and lethal TdP occurring even during short episodes of anesthesia (Odening et al., 2008) (**Figure 6B**).

Transgenic LQT1 rabbits with normal/physiological heart rates and/or without organic heart diseases exhibited relatively homogenous prolongation of repolarization in the LV without substantial increase in APD dispersion; therefore, no spontaneous arrhythmias and SCD were detected (Brunner et al., 2008). When the repolarization reserve of LQT1 rabbits were further challenged by continuous tachypacing or AV-ablation to induce cardiac tachymyopathy (Lau et al., 2015), or complete AV-block (Kim et al., 2015), respectively, however, significant APD dispersion and discordant alternans developed and VT/VF was easily inducible and even occurred spontaneously (Lau et al., 2015). The model also demonstrated the development of pseudo-AV blocks and drug-induced TdP upon *I*_Kr_-blockade by dofetilide and E4031 (Odening et al., 2010; Ziupa et al., 2014; Major et al., 2016; Ziupa et al., 2019) (**Figure 6B**).

The recently generated LQT5 model with slight reduction in *I*_Ks_ function similarly demonstrated more pronounced *I*_Kr_-blocker dofetilide induced TdP formation *in vivo* than healthy WT rabbits (Major et al., 2016) (**Figure 6B**).

#### Limitations of the Currently Available Models and Outlook

As the “dominant-negative” transgenic strategy was used to generate the LQTS rabbits, instead of a rabbit gene “knock-out”/human transgene “knock in” approach; these models are genetically distinct from LQTS patients. Newly emerging genetic engineering techniques such as CRISPR-cas [as described in details in (Bosze et al., 2016)] may help to develop novel—also genetically closer—animal models for these human diseases. As currently available models demonstrate a LQTS phenotype, however, with different degrees of reduction in repolarization reserve and increased susceptibility to arrhythmia both spontaneously and particularly upon drug-induced ion channel blockade, they could still represent valuable models for proarrhythmia safety testing in the context of reduced repolarization reserve.

Furthermore, compensatory electro-mechanical adaptation of cardiomyocytes in response to the altered function (“cardiac remodeling”) can also show species differences that could influence their arrhythmia sensitivity. In transgenic LQTS mice, for example, the observed compensatory upregulation of non-affected potassium currents limit their use in identifying repolarizing prolonging—potentially pro-arrhythmic—agents, as opposed to transgenic LQTS rabbits, in which parallel decrease in reciprocal repolarizing current(s) was seen (Brunner et al., 2008) that even further increased the sensitivity of these models to detect drug-induced proarrhythmia. Currently, very little is known about the nature and extent of these compensatory remodeling processes in human diseases with impaired cardiac repolarization reserve; therefore, it is not known, which animal model mimics most accurately human pathophysiology from this aspect.

In spite of similarities in cardiac ion channels in rabbits and humans, cardiac repolarization is still conveyed through ion channels with slightly different biophysiological characteristics in each species; therefore, caution has to be applied when translating findings based on data from experimental animal models into humans.

Current animal models focus on the detection of repolarization prolonging pro-arrhythmic effects. Drug-induced proarrhythmia may, however, also occur upon pathologically pronounced acceleration/shortening of cardiac repolarization (Malik, 2016). Here, novel models such as the transgenic short QT syndrome rabbit model (Odening et al., 2019), may be particularly useful for the detection of these pro-arrhythmic drug properties.

Further improvement of current *in silico* proarrhythmia screening capabilities by integrating experimental *in vivo*, whole heart, cellular, and ion channel data into computational models are highly warranted (Sager et al., 2014; Colatsky et al., 2016), and could potentially lead to better assessment of mutation-specific aspects of a wide range of cardiac channelopathies, since currently available animal models mimicking cardiac channelopathies are very limited.

Importantly, the future use of patient-specific and/or disease-specific human induced pluripotent stem cell derived cardiomyocytes (iPSC-CM) has the potential to become a relevant additional patient/disease-specific *in vitro* safety screening platform to test the pro-arrhythmic potential of novel drug candidates (Mehta et al., 2013; Sager et al., 2014; Colatsky et al., 2016).

## Author Contributions

IB wrote the manuscript and designed the figures. TH wrote the manuscript and designed the figures. MB and GK generated the LQT1 and LQT2 rabbits and edited the manuscript. KO wrote the manuscript and designed the figures.

## Funding

This work was supported by a grant from the German Heart Foundation (F/02/14) and grants from the German Research Foundation (OD 86/6-1, and BR 2107/4-1) to KO and the Hungarian National Research, Development and Innovation Office (NKFIH K128851) and Ministry of Human Capacities (EFOP-3.6.2-16-2017-00006) to IB.

## Conflict of Interest

The authors declare that the research was conducted in the absence of any commercial or financial relationships that could be construed as a potential conflict of interest.
